# Evaluation of somatostatin, CXCR4 chemokine and endothelin A receptor expression in a large set of paragangliomas

**DOI:** 10.18632/oncotarget.21194

**Published:** 2017-09-23

**Authors:** Daniel Kaemmerer, Jörg Sänger, Ruza Arsenic, Jan G. D’Haese, Jens Neumann, Annette Schmitt-Graeff, Ralph Markus Wirtz, Stefan Schulz, Amelie Lupp

**Affiliations:** ^1^ Department of General and Visceral Surgery, Zentralklinik Bad Berka, Bad Berka, Germany; ^2^ Laboratory of Pathology and Cytology Bad Berka, Bad Berka, Germany; ^3^ Institute of Pathology, Charité University Hospital Berlin, Berlin, Germany; ^4^ Department of General, Visceral, Transplantation, Vascular and Thoracic Surgery, Hospital of the University of Munich, Munich, Germany; ^5^ Department of Pathology, Ludwig-Maximilians-University Munich, Munich, Germany; ^6^ Department of Pathology, Albert-Ludwigs-University of Freiburg, Freiburg, Germany; ^7^ STRATIFYER Molecular Pathology GmbH, Köln, Germany; ^8^ Institute of Pharmacology and Toxicology, Jena University Hospital, Jena, Germany

**Keywords:** somatostatin receptors, CXCR4, endothelin receptor A, immunohistochemistry, paraganglioma

## Abstract

Paragangliomas are predominantly benign tumors, but in some cases invasive growth and also metastasis are observed. Given the limited number of nonsurgical treatment options, novel target structures for diagnostics and therapy of this tumor entity are urgently needed.

In the present study, expression of all five somatostatin receptor (SST) subtypes, chemokine receptor CXCR4 and endothelin receptor type A (ETA) was assessed by means of immunohistochemistry in a total of 66 paraffin-embedded paraganglioma samples from 55 patients. The stainings were rated by means of the Immunoreactive Score and correlated to clinical data and to succinate dehydrogenase subunit B (SDHB) expression.

SST2A was by far the most prominent receptor in the paragangliomas investigated. It was present in 89% of the tumors at a high intensity, followed by SST5, SST3, SST1 and SST4, which were detected in 47%, 35%, 35% and 13% of the samples, respectively. SDHB positive tumors exhibited significantly higher SST2A and SST3 expression as compared to SDHB negative cases. There was no correlation between SST and Ki-67 expression or grading of the tumors and no difference in SST expression between primary tumors and metastases. Cell surface expression of CXCR4 and ETA was detected only in few samples. On tumor capillaries, however, exceptionally strong staining for these two receptors was noticed in the vast majority of the tumors.

In conclusion, paragangliomas are well suited for SST2A-based diagnostics and treatment modalities. An indirect targeting of these highly vascularized tumors via CXCR4 or ETA may also represent a promising future strategy.

## INTRODUCTION

Paragangliomas are rare, highly vascularized neuroendocrine tumors, which derive from the embryonic neural crest and are closely related to pheochromocytomas [1]. While thoracic and abdominal paragangliomas arise from sympathetic-lineage derived cells and are often associated with catecholamine overproduction, paragangliomas of the head and the neck are of parasympathetic origin and usually do not secrete catecholamines [2, 3]. About 25-50% of the paragangliomas are hereditary, resulting from germline mutations in different tumor predisposition genes, as e.g. in the genes of the different succinate dehydrogenase (SDH) subunits, leading to an increased malignant potential of these tumors [4–11]. Especially SDHB mutations hve been shown to be associated with metastatic disease in paragangliomas and according to literature data [5, 6, 10] about half of the patients with metastatic paragangliomas (especially those of sympathetic origin) display SDHB mutation. Paragangliomas are predominantly benign, but depending on the size, localization, and genetic background approximately 10-50% can develop metastases e.g. into lymph nodes, liver, lung, and bone [3, 8, 12, 13]. The only effective treatment of paragangliomas is surgical resection, and patients with inoperable tumors die from metastatic disease or from cardiovascular complications due to the excess catecholamine production seen with some of the tumors. Nonsurgical treatment options are limited and thus identification of novel target structures allowing for diagnostics and for pharmaco- or radiotherapy of these tumors is urgently needed. However, respective studies are rare, probably also because of the scarcity of these tumors.

Neuroendocrine tumors are well known for their over-expression of somatostatin receptors (SST), especially of SST2A, but also SST5, which represent the molecular basis of different SST-based diagnostic and treatment modalities. Thus, a number of studies have already been performed assessing SST expression also in paragangliomas, both at the mRNA and at the protein level [14–20]. These studies yielded, however, quite different results with respect to the expression pattern and expression rate of individual receptor subtypes in this tumor entity (Table 1). This may be due to the generally small sample size and the different methods and techniques used, but may also have been influenced by the type of antibody (polyclonal vs. monoclonal) employed in the immunohistochemical investigations. There may also be differences between tumors of parasympathetic or sympathetic origin, between benign tumors and metastatic disease or between hereditary and non-hereditary neoplasms. For instance, recently higher SST2A and SST3 expression levels have been shown in SDHB-deficient as compared to SDHB positive tumors [20].

**Table 1:** Studies on somatostatin receptor expression

Authors (method)	Tumors	SST2A	SST3	SST5
**Elston et al. (2015) (IHC)**	Pheo/PGL	SDHB-deficient tumors: 91% moderate-strong expression, SDHB-sufficient tumors: 49% expression	SDHB-deficient tumors: 50 % moderate-strong expression, SDHB-sufficient tumors: 21% expression	SST5 very low expression
**Saveanu et al. (2011) (IHC, RT-PCR)**	Pheo/PGL (SDHx mutation: no data)	SST2A >> SST1,3,4,5	-	SST5 low expression
**Kimura et al. (2010) (IHC)**	Familial PGL (SDHD positive)	SST2A positive	-	SST5 negative
**Kölby et al. (2006) (RT-PCR)**	Pheo/PGL (SDHx mutation: no data)	SST2A abundant expression	-	-
**de Herder and Hofland (2004) (Review)**	Pheo	SST2A 87% positive expression	-	-
**Mundschenk et al. (2003) (IHC)**	Pheo (SDHx mutation: no data)	SST2A 25% positive expression	SST3 90% positive expression	SST5 low expression
**Epelbaum et al. (1995) (RT-PCR)**	Pheo/PGL (SDHx mutation: no data)	SST2A expression	SST3 expression	SST5 expression
**Reubi et al. (1992) (Autoradiogr.)**	Pheo/PGL (SDHx mutation no data)	Presence of somatostatin binding sites but no SST receptor subtype differentiation		

IHC: immunohistochemistry; Autoradiogr.: *in vitro* autoradiography; SST: somatostatin receptor; Pheo: pheochromocytomas; PGL: paragangliomas

The CXCR4 is a plasma membrane chemokine receptor, which is physiologically involved in organogenesis, hematopoiesis and inflammation [21, 22]. Additionally, CXCR4 over-expression has been shown in more than 20 different tumor entities and increased CXCR4 expression has been associated with rapid tumor progression, high invasiveness, early metastasis and poor patient outcome. Furthermore, it has been demonstrated that an increased CXCR4 expression is associated with advanced tumor stage and poor patient outcome also in neuroendocrine tumors [23–26]. In the meantime, several CXCR4 antagonists have been synthesized [27], some of which like AMD3100 (plerixafor) have even been evaluated for their therapeutic potential in cancer patients [28]. Besides, radiolabelled CXCR4 ligands, such as the Ga-68 labelled receptor ligand CPCR4-2, have been shown to be excellently suited for CXCR4-based PET diagnostics, especially in highly proliferative tumor entities [29]. However, although many types of tumors have been studied for the expression of CXCR4 [30], no respective investigations have been performed with (malignant) paragangliomas so far.

As paragangliomas are highly vascularized tumors, an alternative treatment option by targeting of the tumor vessels has also been suggested. It has been shown that part of the tumors, especially those with a mutation in the SDHB gene, display an increased expression of several important pro-angiogenic factors such as VEGF and its receptors, HIF2alpha, angiopoietin-2, endothelin-1 and the endothelin receptors ETA and ETB [31]. Among the three different endothelin isoforms, ET-1 is the predominant one. It acts (via ETA) as a potent vasoconstrictor and is known to be involved in a variety of cardiovascular and renal disorders. Besides, the ET-1/ETA axis has also been shown to play an important role in the development of cancer. Here, it acts through activation of pathways involved in cell proliferation, escape from apoptosis, invasion, metastasis, epithelial-mesenchymal transition, immune modulation, aberrant osteogenesis and angiogenesis [32–35]. Although identified as promising target structures also in neuroendocrine tumors such as medullary thyroid carcinoma or pituitary adenoma [36, 37], there are only two studies available so far examining the expression pattern of different endothelins and their receptors in paragangliomas [31, 38].

In view of the contradictory results in literature, in the present study the SST expression pattern was re-evaluated in a large set of paragangliomas, differentiating between parasympathetic and sympathetic tumors, primary tumors and metastases, and SDHB positive and negative cases. We have focused on SDHB mutations since of all SDHx mutations the prevalence for this mutation is high in paragangliomas and since it predisposes to malignancy. SDHA and SDHC mutations are much less frequently present in paragangliomas, and SDHD mutations are usually associated with benign disease [3, 7, 8, 10–12]. To the best of our knowledge, this study represents the most comprehensive and largest study on SST subtypes expression in paragangliomas so far. In addition, the chemokine receptor CXCR4 and the endothelin receptor A (ETA) were evaluated for their suitability as novel diagnostic and therapeutic targets in paragangliomas. Receptor expression was assessed by means of immunohistochemistry (IHC) using novel rabbit monoclonal antibodies against the SSTs, CXCR4, and ETA. These antibodies, displaying numerous advantages over polyclonal ones, have been generated by our group against the respective carboxyl-terminal tails of the receptors and extensively characterized recently [37, 39–43]. To further strengthen the IHC results, SSTR expression was additionally evaluated at the mRNA level by means of qRT-PCR in serial paraffin sections of a small subset of the samples.

## RESULTS

### Patient characteristics

**Figure 1:**
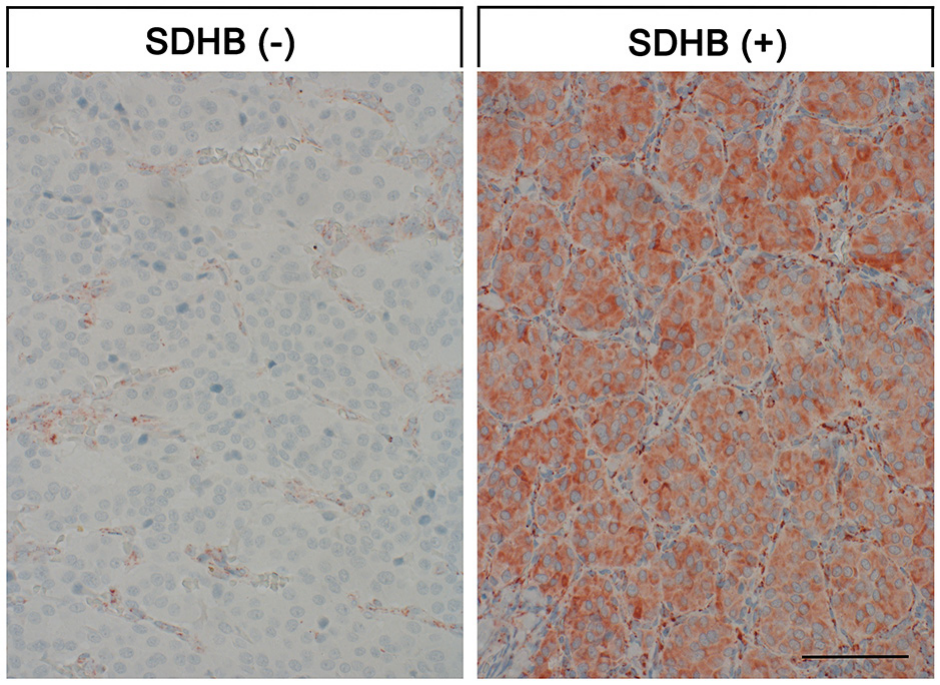
SDHB expression in paragangliomas Depicted are typical examples for SDHB-negative and SDHB-positive samples. Scale bar, 50 μm.

In the present study, in total 66 FFPE from 55 patients with histologically verified paraganglioma were evaluated. Pheochromocytomas were excluded from the present investigation. All patients were Caucasians. From the patients, 13 were male, 21 female, and from 21 the gender was unknown. The 55 tumor samples comprised 45 primary tumors and 10 metastases (4 lymph node, 1 pulmonary, 2 intraspinal, 3 bone metastases), 3 samples were from relapses. 31 tumors originated from parasympathetic and 20 from sympathetic ganglia, from 4 tumors the provenance was unknown. Of the parasympathetic tumors the localization of the primary tumors was as follows (n): skull base (2); nasal passage and maxillary sinus (3); pharynx (1); glomus tympanicum (2); glomus caroticum (19); glomus jugulare (4). Of the sympathetic tumors, the provenance was as follows (n): mediastinum (4); abdominal aorta (5); intraspinal (2); infrarenal or close to kidney (3); close to jejunum (2); close to adrenals (1); close to gallbladder (1); close to urinary bladder (1); pelvis (1). According to the immunohistochemical SDHB stainings, 41 tumors were SDHB positive and 12 SDHB negative (including the two tumors with known SDHB mutation) (for typical staining results see Figure 1). In 2 samples a clear-cut assignment was not possible for technical reasons. The mean age of the patients at diagnosis was 47.8 years (median 47.4, range 13.0 - 80.0 years), with no major difference between the SDHB positive and negative cases (median age: 45.7 vs. 49.0 years). In two patients a mutation in the SDHB gene has been verified by genetic testing beforehand. At diagnosis these patients were 22 and 25 years old.

### Somatostatin receptor expression

**Figure 2:**
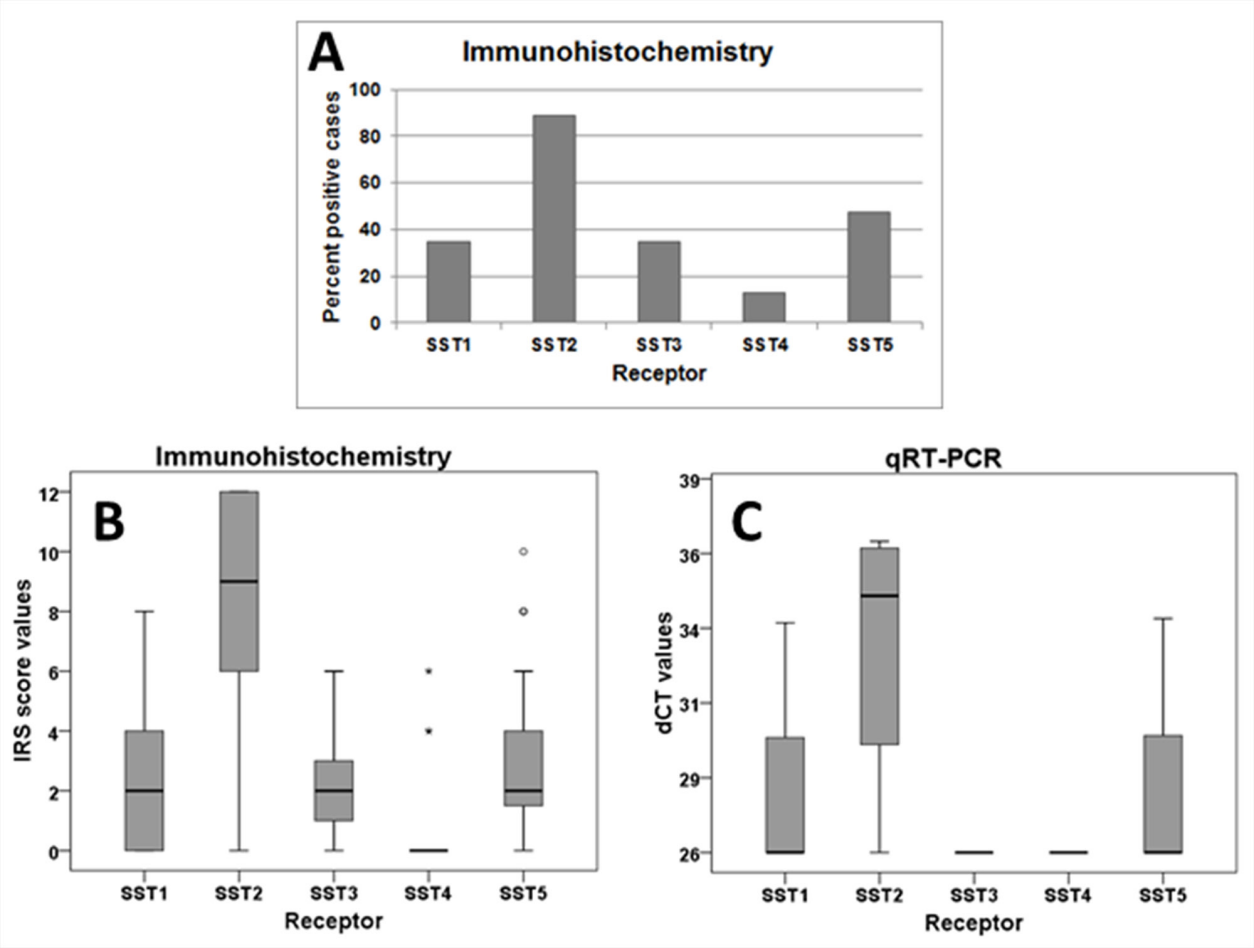
Frequency distribution and intensity of expression of SST subtypes in paragangliomas **(A)**, Number of positive cases for different SSTs in immunohistochemical stainings. **(B)**, Box plots of expression levels (IRS values) of SSTs as determined by immunohistochemistry. Depicted are the median of mean patient values, upper and lower quartiles, minimum and maximum values, and outliers. **(C)**, Box plots of expression levels (dCT-values) of SSTs as determined by qRT-PCR.

SST2A was by far the most prominently expressed receptor in the paraganglioma samples investigated (Figure 2A). It was present in 89.1% of the cases at a high intensity (median IRS: 9 points; Figure 2B), followed by SST5, SST1, SST3, and SST4, which were detected in 47.3%, 34.5%, 34.5% and in 12.7% of the tumors at a much lower intensity of expression (median IRS of SST1, SST3 and SST5: 2 points, SST4: 0 points; Figure 2B). A very similar pattern was observed in the qRT-PCR analysis of receptor mRNA expression (Figure 2C).

**Figure 3:**
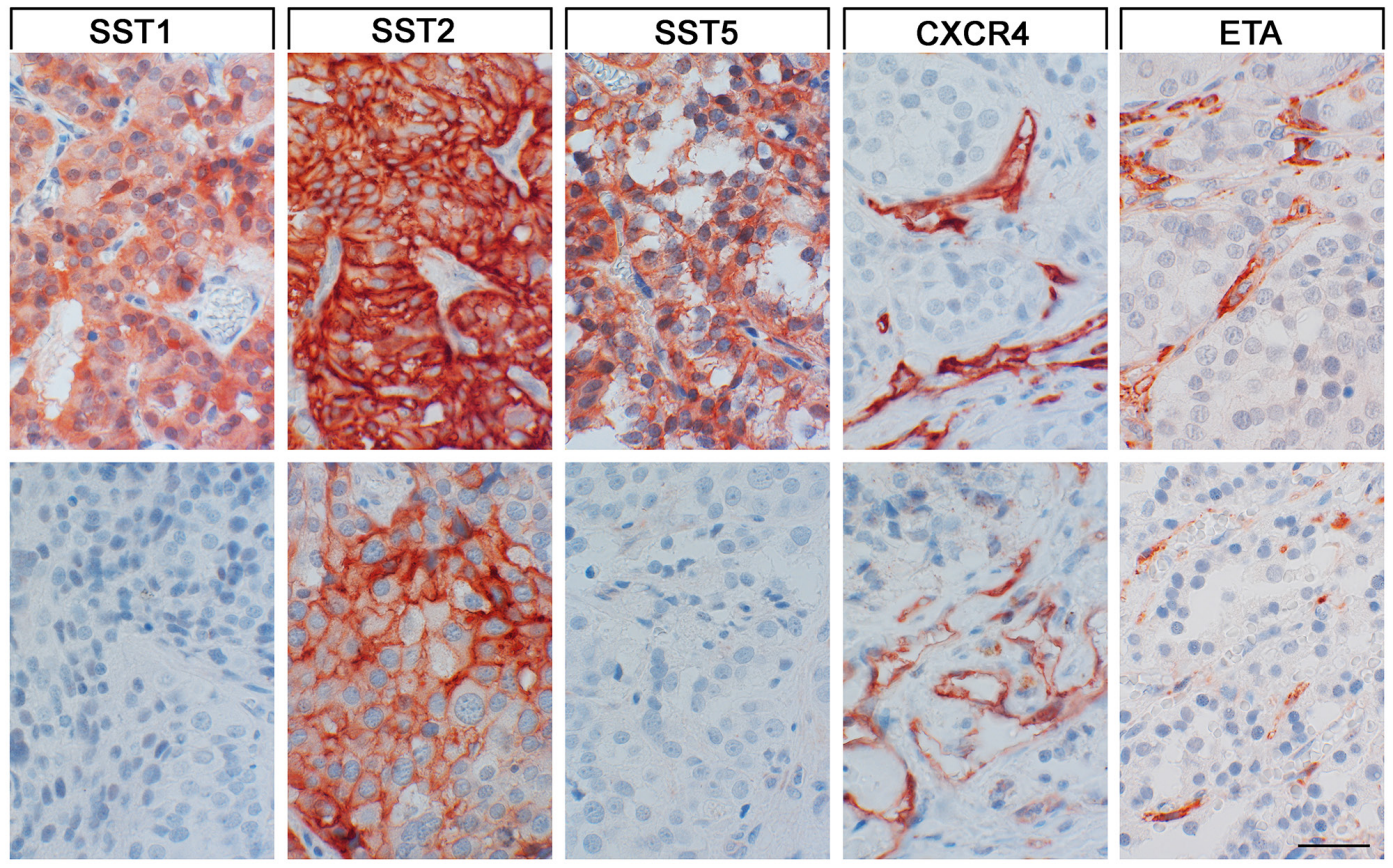
SST, CXCR4 and ETA expression in paragangliomas Depicted are two typical examples each for the staining patterns of the SST1, SST2A, SST5, CXCR4, and ETA. Scale bar, 50 μm. Top row of photomicrographs: SST1, SST2A, SST5: metastasis of an intraspinal sympathetic SDHB positive tumor; CXCR4, ETA: lymph node metastasis of a pelvic sympathetic SDHB negative tumor; bottom row of photomicrographs: SST1, SST2A, SST5: lymph node metastasis of a pelvic sympathetic SDHB negative tumor; CXCR4: primary mediastinal sympathetic SDHB negative tumor; ETA: metastasis of an intraspinal sympathetic SDHB positive tumor.

As can be seen from Figure 3, in all cases immunostaining for SST2A was mainly localized at the plasma membrane of the tumor cells, whereas for SST1, SST3 and SST5 both a cytoplasmic and a membraneous staining was observed. For SST4, in contrast, only cytoplasmic immunoreactivity was found. Besides, tumor capillaries stained positive for SST2A in 69.2%, and for SST5 in 64.7% of the cases.

**Figure 4:**
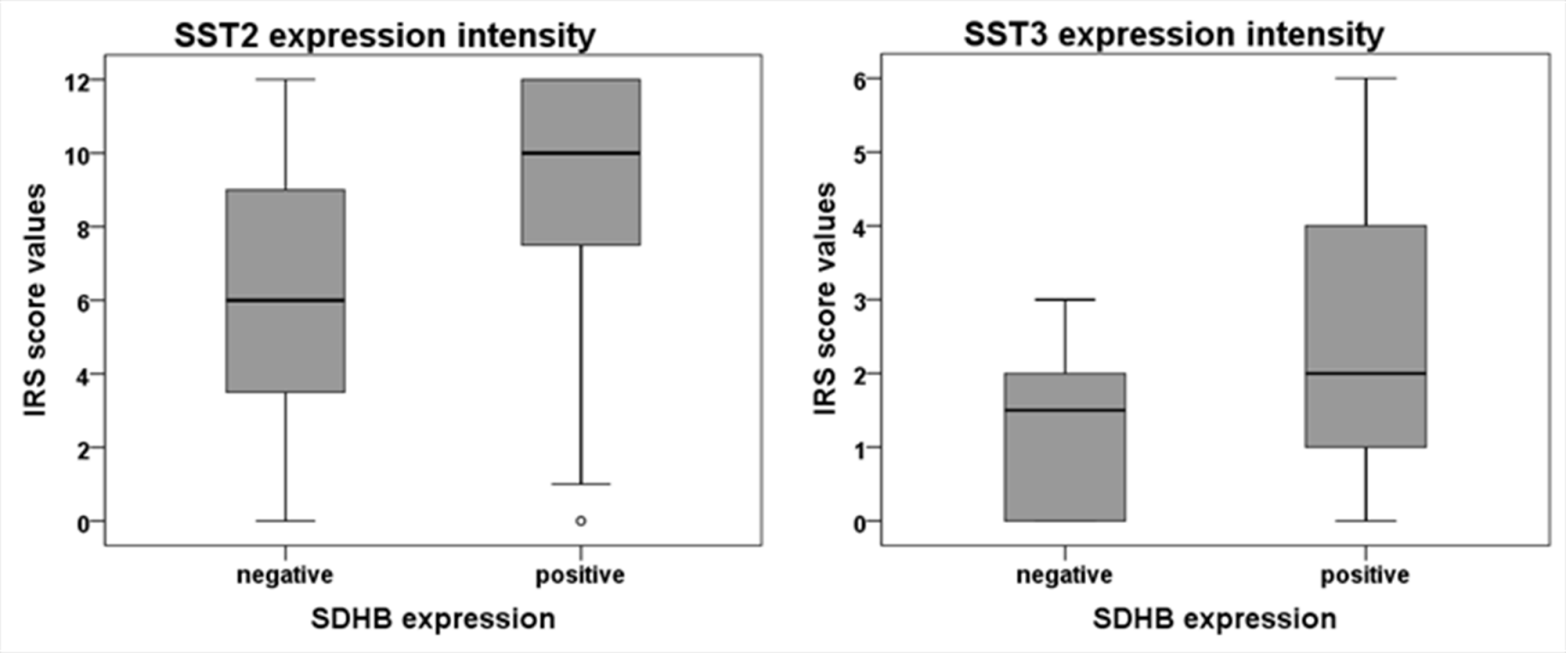
SST2A and SST3 expression intensity in SDHB-negative and -positive paragangliomas Box plots of expression levels (IRS values) of SST2A and SST3 as determined by immunohistochemistry. Depicted are the median of mean patient values, upper and lower quartiles, minimum and maximum values, and outliers.

At the protein level, significant correlations were seen between expression of SST1 and SST5, between SST2A and SST5, and between SST3 and SST5 expression in the tumor cells (Table 2). There was no correlation between SST and Ki-67 expression values or the grading of the tumors. Metastases displayed significantly higher Ki-67 values as compared to the primary tumors (p=0.028), but there were no significant differences with respect to SST expression. Sympathetic tumors were associated with a lower patient age (p=0.091) and higher Ki-67 values (p=0.014) as compared to parasympathetic tumors. SDHB negative tumors exhibited significantly lower SST2A score values (median: 6 vs. 10 IRS points; p=0.021) and SST3 score values (median: 1.5 vs. 2 IRS points; p=0.027) (Figure 4) and also significantly fewer SST3 positive cases (1/12 cases vs. 18/41; p=0.024), but higher Ki-67 values (median: 5% vs. 2%; mean: 13.2% vs. 3.4%; p=0.003) as compared to SDHB positive tumors. While 35.7% of the sympathetic tumors were SDHB negative, among parasympathetic tumors only 19.2% were SDHB negative. This difference, however, was not statistically significant. There was no correlation between SST expression and patient age at initial diagnosis. In comparison to male patients, females had significantly higher SST2A and SST3 IRS values (median SST2A: 7 vs. 12 IRS points; p=0.018; median SST3: 1 vs. 3 IRS points; p=0.026), which was mirrored by a higher percentage of SST3 positive tumors (p=0.059), too. 59.3% of female patients, but only 25.0% of the male patients had parasympathetic tumors (which are more often SDHB positive and show lower Ki-67 values) (p=0.048).

**Table 2:** Correlation between expression intensities of different SSTs and Ki-67 in all paraganglioma cases investigated

		SST2A	SST3	SST4	SST5	Ki-67
**SST1**	r	0.122	0.260	0.197	**0.434**	0.296
	p	0.373	0.055	0.150	**0.001**	0.051
**SST2A**	r		0.182	0.065	**0.324**	-0.079
	p		0.184	0.638	**0.016**	0.604
**SST3**	r			-0.041	**0.451**	-0.019
	p			0.769	**0.001**	0.901
**SST4**	r				-0.020	-0.027
	p				0.882	0.860
**SST5**	r					0.120
	p					0.432

r: correlation coefficient (Spearman); p: p value

### CXCR4 chemokine and endothelin A receptor expression

CXCR4 and ETA were detected only in a few cases on the tumor cells. Tumor cell-associated expression of CXCR4 was present in 9 samples, whereas ETA expression was observed in 3 cases only. Here, the median IRS for CXCR4 amounted to 2 points and for ETA to 0 points. Thus, if present, the intensity of expression was only weak. Staining for CXCR4 and ETA was predominantly detected on the plasma membrane of the positive tumor cells. Most notably and in contrast to the scarce staining of tumor cells, exceptionally strong staining of tumor capillaries was noticed for both ETA (89.5% of cases) and CXCR4 (73.7% of cases) (Figure 3). Besides, ETA was also expressed at higher levels on fibroblasts and fibrocytes of the tumor stroma. There were no correlations between ETA or CXCR4 stainings of the tumor cells or the percentages of ETA- or CXCR4-positive tumor capillaries and SST, Ki-67, SDHB expression or other clinical data of the patients.

## DISCUSSION

### Somatostatin receptor expression

In our study, SST2A was the most significantly expressed SST in the paragangliomas investigated, followed by SST5 and the other SSTs, which were clearly of secondary importance. These results were further supported by qRT-PCR data. In previous investigations of paragangliomas quite different findings with respect to the expression pattern and the intensity of expression of individual SST subtypes were reported. However, similar to our investigations, in all these studies either a predominance or at least a high expression level of SST2A was observed [15–20]. Contradictory results were mainly reported with respect to other SST subtypes, which may be due to different methods and techniques used (autoradiography and PCR vs. immunohistochemistry) and the type of antibody employed in the immunohistochemical investigations. With the only exception of the study of Elston et al. [20], previous investigations used polyclonal antibodies for detection of SSTs. In the present study a lower SST2A and SST3 intensity and also a lower SST3 expression frequency was observed in SDHB-negative cases. These results are in contrast to the findings of Elston et al. [20], who reported higher SST2A and SST3 expression rates in SDHB negative tumors, using the same antibody for the differentiation between SDHB-positive and -negative cases as in the present investigation. SDHB-negative paragangliomas are reported to be of higher malignancy [4–11], which is mirrored also in our investigation by the significantly higher Ki-67 values of SDHB-negative tumors. This finding indirectly strengthens our SDHB staining results. Additionally, it has been shown in gastroenteropancreatic neuroendocrine neoplasms that, with increasing malignancy, SST2A expression is significantly decreased [24]. Our current results appear to support this correlation.

Overall, the generally high expression rate of the SST2A found in the present investigation even in SDHB negative cases suggests that paragangliomas are well suited for SST2A-based therapies and diagnostics, regardless of the genetic background or location of the tumor or the presence of metastatic disease. Since the other SST subtypes are expressed at significantly lower levels, an advantage of pan-somatostatin analogs, e.g. pasireotide, is not to be expected. This view is supported by the good sensitivity and specificity observed in many studies with respect to scintigraphy and PET/CT imaging using radiolabeled somatostatin analog derivatives also in SDHB negative tumors (see e.g. [48–63]). Likewise, treatment of inoperable paragangliomas with octreotide [64–67] or by means of SST-based radionuclide therapy [68–71] yielded very promising results with a prolonged overall survival of patients with metastatic disease. The wide SST2A distribution with a strong receptor expression intensity found in the present study in paragangliomas provides the basis and rationale of this clinically successful treatment.

### CXCR4 chemokine and endothelin A receptor expression

A strong expression of CXCR4 on tumor vessels has already been shown for other tumor entities and, hence, targeting of these vessels via CXCR4 has already been suggested as a novel cancer therapy [72]. In our study, both CXCR4 and ETA were found to be intensely expressed on tumor vessels of the paragangliomas investigated. This is in good accordance with the findings of the only other study, in which ETA expression has been evaluated in the same tumor entity [38]. Since paragangliomas are highly vascularized tumors, they represent good candidates for an antiangiogenic therapy [31]. Thus, although CXCR4 and ETA are hardly expressed by the tumor cells themselves, both receptors clearly enable an indirect targeting of the tumors via their blood vessels, thus depriving them of their supportive environment. Targeting of the tumor stroma represents an interesting therapeutic strategy, which is gaining increasingly attention in the literature [72].

## CONCLUSION

Due to the high SST2A expression level found in the present study, paragangliomas seem to be well suited for SST2A-based diagnostics and therapies. Since the other SSTRs were present at much lower levels, pan-somatostatin analogs appear to be less appropriate to control tumor progression, but SST2A-selective drugs may benefit from a limited side-effect profile. Indirect targeting of these highly vascularized tumors via CXCR4 or ETA may also represent a promising therapeutic strategy.

## MATERIALS AND METHODS

### Tumor specimens

In the present investigation, 66 archived formalin-fixed-paraffin-embedded (FFPE) tumor samples from 55 patients were included (in detail, 49×1, 4×2, 1×3, and 1×6 samples per patient). The samples had been removed by surgery between 2004 and 2015 and were histopathologically verified by two independent experienced pathologists as paragangliomas. The samples were provided by the Institute of Pathology and Cytology, Bad Berka, Germany, by the Institute of Pathology, Charité University Hospital Berlin, Germany, by the Department of Pathology, Ludwig-Maximilians-University Munich, Germany, and by the Department of Pathology, Albert-Ludwigs-University of Freiburg, Germany. If available, clinical data were gathered from the patient records. All procedures performed in this study involving human participants were in accordance with the 1964 Helsinki declaration and its later amendments. Permission was gained from the local ethics committee (Ethikkommission der Landesärztekammer Thüringen, Germany) for this retrospective analysis. All data were recorded and analyzed anonymously.

### Immunohistochemistry

From the paraffin blocks 4 μm sections were prepared and floated onto positively charged slides. Immunostaining was performed by an indirect peroxidase labeling method as described previously [44]. Briefly, sections were dewaxed, microwaved in 10 mM citric acid (pH 6.0) for 16 min at 600 W and then incubated with the specific primary antibodies overnight at 4°C. For the detection of SSTs (except SST4), CXCR4 and ETA novel rabbit monoclonal antibodies were used (hybridoma cell culture supernatants), which are directed against the respective carboxyl-terminal tail of the different receptors and which have been extensively characterized previously ([37, 39–43]; for detailed information on the clones, epitopes and the dilutions of the antibodies used see Table 3). With respect to SST4, similar but polyclonal antibodies (Gramsch Laboratories, Schwabhausen, Germany) were applied. SST2 exists in two splice variants, SST2A and SST2B, which differ in their carboxyl-terminal tail. In contrast to rodents, SST2B is not present in human tissues [39]. Therefore, only SST2A expression was assessed in the present investigation, which is selectively detected by the antibody UMB-1 (Table 3). Sections from normal human pancreas (islets; SST1, SST2A, SST3, SST5), lymph nodes (germinal centers; SST2A, SST5, CXCR4), human cortex (SSTR4), and human heart atria (ETA) served as positive controls. As negative control, the primary antibody was either omitted or adsorbed for 2 h at room temperature with 10 μg/ml of the peptide used for immunizations. In all cases a complete abolition of immunostaining was observed. Additional stainings were performed with monoclonal mouse antibodies against the proliferation marker Ki-67, chromogranin A, as a marker for neuroendocrine tissue, CD34, as a marker for tumor vessels and SDHB (Table 3). In the SDHB stainings, two patients with known genetic SDHB mutation served as negative control. Detection of the primary antibodies was performed using a biotinylated anti-rabbit or anti-mouse IgG, respectively, followed by an incubation with peroxidase-conjugated avidin (Vector ABC “Elite” kit; Vector, Burlingame, CA; dilution: 1:200). Binding of the primary antibodies was visualized using 3-amino-9-ethylcarbazole (AEC) in acetate buffer (BioGenex, San Ramon, CA; dilution 1:5). Sections were then rinsed, counterstained with Mayer's hematoxylin (Sigma-Aldrich Chemie GmbH, Steinheim, Germany) and mounted in Vectamount™ mounting medium (Vector Laboratories, Burlingame, CA).

**Table 3:** Antibodies used for immunohistochemical stainings

Antibody	Clone	Type	Epitope	Supplier	Dilution
**SST1**	UMB-7	rabbit monoclonal	ENLESGGVFRNGTCTSRITTL(residues 377-391)	Epitomics, Burlingame, CA	1:25
**SST2A**	UMB-1	rabbit monoclonal	ETQRTLLNGDLQTSI(residues 335-369)	Epitomics, Burlingame, CA	1:10
**SST3**	UMB-5	rabbit monoclonal	QLLPQEASTGEKSSTMRISYL(residues 398-418)	Epitomics, Burlingame, CA	1:20
**SST4**	4802	rabbit polyclonal	CQQEALQPEPGRKRIPLTRTTIF(residues 366-388)	Gramsch, Schwabhausen, Germany	0.1 μg/ml
**SST5**	UMB-4	rabbit monoclonal	QEATPPAHRAAANGLMQTSKL(residues 344-364)	Epitomics, Burlingame, CA	1:10
**CXCR4**	UMB-2	rabbit monoclonal	KGKRGGHSSVSTESESSSFHSS(residues 338-359)	Epitomics, Burlingame, CA	1:2
**ETA**	UMB-8	rabbit monoclonal	KNHDQNNHNTDRSSHKDSMN(residues 408-427)	Epitomics, Burlingame, CA	1:10
**Ki-67**	MIB-1	mouse monoclonal		DAKO, Hamburg, Germany	1:75
**CgA**	LK2H10	mouse monoclonal		biologo, Kronshagen,Germany	1:50
**CD34**	QBEnd 10	mouse monoclonal		DAKO, Hamburg, Germany	1:50
**SDHB**	21A11AE7	mouse monoclonal		Abcam, Milton, UK	1:500

CgA: Chromogranin A

The stainings for the receptors of all sections were scored by means of the semiquantitative Immunoreactivity Score (IRS) according to Remmele and Stegner [45], as modified by McCarty et al. [46], multiplying the percentage of positive tumor cells in five gradations (no positive cells, 0; <10% positive cells, 1; 10-50% positive cells, 2; 51-80% positive cells, 3; >80% positive cells, 4) with the staining intensity in four gradations (no staining, 0; mild staining, 1; moderate staining, 2; strong staining, 3). As a result, score values between 0 and 12 were obtained. In case that one patient had more than one tumor slide, an arithmetic mean was calculated from the IRS of each slide. Samples, having values ≥3 IRS points were considered positive. Staining of the tumor vessels for SST2A, SST5, CXCR4 and ETA was evaluated separately by determining the percentage of positive vessels in relation to all vessels showing CD34-positivity. Tumors with >10% of the CD34 positive vessels being stained for the respective receptor were considered positive. With respect to Ki-67 staining, the percentage of positive nuclei was determined.

### Quantitative real-time-polymerase-chain-reaction (qRT-PCR)

In cooperation with STRATIFYER Molecular Pathology, Cologne, Germany, one adjacent paraffin section from the immunohistochemical slides from 4 patients, which were randomly and blindly selected, were processed fully automatically to measure SST mRNA expression. Sufficient mRNA was isolated from these FFPE specimens using a standardized isolation method, based on magnetic beads [Extraction-XL (96) RNA 2.0 kit; STRATIFYER Molecular Pathology, Köln, Germany] as previously described [47]. Directly after extraction, multiplex TaqMan-real-time PCR was performed fully automated using the SuperScript III Platinum One-Step real-time RT-PCR kit and Platinum Taq DNA polymerase (Invitrogen, Darmstadt, Germany). Primers and probes for the SST subtypes were designed by STRATIFYER Molecular Pathology and prepared by Eurogentec (Liège, Belgium). The housekeeping gene CALM2 (calmodulin 2) was used as a control. Additionally, a no-template control (NTC) and a human reference RNA (Agilent Technologies, Santa, Clara CA) were both analyzed in parallel. As external controls, slides from gastroenteropancreatic neuroendocrine tumors with known high SST expression were used. Measurements were conducted on an Mx3005P device using the MxPro version 4.10d software (Agilent Technologies, Santa Clara, CA). After 40 cycles (50 min at 30°C, 2 min at 95°C [15 s at 95°C, 45 s at 60°C] x 40), a logarithmic analysis was performed at a threshold of 50. Values were normalized using the following formula for cycle threshold (Ct): dCt(norm) = 40 - DCt, with DCt = Ct(SSTR) - Ct(CALM2). As a result, dCt values ≥19.00 were obtained and used for further calculations.

### Statistics

For statistical analysis, the IBM SPSS statistics program version 22.0.0.0 was used. Because the data were not normally distributed except for the age of the patients (Kolmogorov-Smirnov test), Mann-Whitney test, Spearman's rank correlation and Kendall's τ-b test as well as χ^2^ test were performed. A p value ≤0.05 was considered significant.

## Abbreviations

Ct: cycle threshold
ETA: endothelin receptor A
IHC: immunohistochemistry
SST: somatostatin receptor
